# Use of urinary 13,14, dihydro-15-keto-prostaglandin F_2α_ (PGFM) concentrations to diagnose pregnancy and predict parturition in the giant panda (*Ailuropoda melanolecua*)

**DOI:** 10.1371/journal.pone.0195599

**Published:** 2018-05-02

**Authors:** Beth M. Roberts, Janine L. Brown, David C. Kersey, Rebecca J. Snyder, Barbara S. Durrant, Andrew J. Kouba

**Affiliations:** 1 Department of Research and Conservation, Memphis Zoo, Memphis, Tennessee, United States of America; 2 Center for Species Survival, Smithsonian Conservation Biology Institute, Front Royal, Virginia, United States of America; 3 College of Veterinary Medicine, Western University of Health Sciences, Pomona, California, United States of America; 4 Department of Mammals, Zoo Atlanta, Atlanta, Georgia, United States of America; 5 Institute for Conservation Research, San Diego Zoo Global, San Diego, California, United States of America; Sichuan University, CHINA

## Abstract

Pregnancy determination is difficult in the giant panda (*Ailuropoda melanolecua*), representing a challenge for *ex situ* conservation efforts. Research in other species experiencing pseudopregnancy indicates that urinary/fecal concentrations of 13,14, dihydro-15-keto-prostaglandin F_2α_ (PGFM) can accurately determine pregnancy status. Our objective was to determine if urinary PGFM concentrations are associated with pregnancy status in the giant panda. Urinary PGFM concentrations were measured in female giant pandas (n = 4) throughout gestation (n = 6) and pseudopregnancy (n = 4) using a commercial enzyme immunoassay. Regardless of pregnancy status, PGFM excretion followed a predictable pattern: 1) baseline concentrations for 11–19 weeks following ovulation; 2) a modest, initial peak 14–36 days after the start of the secondary urinary progestagen rise; 3) a subsequent period of relatively low concentrations; and 4) a large, terminal peak at the end of the luteal phase. Pregnant profiles were distinguished by an earlier initial peak (*P* = 0.024), higher inter-peak concentrations (*P* < 0.001), and a larger terminal peak (*P* = 0.003) compared to pseudopregnancy profiles. Parturition occurred 23 to 25 days from the initial PGFM surge and within 24 hours of the start of the terminal increase. These pattern differences indicate that urinary PGFM monitoring can be used to predict pregnancy status and time parturition in the giant panda. Furthermore, this is the only species known to exhibit a significant PGFM increase during pseudopregnancy, suggesting a unique physiological mechanism for regulating the end of the luteal phase in the giant panda.

## Introduction

Once widespread throughout southern China, only ~2,000 giant pandas remain in the wild [1], and these populations are distributed across a highly fragmented habitat [2]. Captive giant pandas represent ~20% of the global population and are thus a major resource for the species’ survival [3]. Intensive research over the last 30 years has improved giant panda husbandry and assisted breeding techniques, resulting in a sustainable captive population [3–6]. Yet, the lack of reproductive success in some captive individuals remains a challenge for preserving long-term genetic diversity [3, 7]. Considerable research has been conducted on the biology of giant pandas (*Ailuropoda melanolecua*), but many aspects of this species’ reproductive physiology remain poorly understood, including biomarkers of pregnancy and pregnancy loss. The lack of a definitive pregnancy diagnosis tool presents a major challenge for *ex situ* species management. Thus, a better understanding of the unusual and complex reproductive physiology of this species would benefit conservation efforts.

Studies on wild giant pandas in the mid-80s revealed that this bear species is seasonally monestrous, breeds in late winter or early spring, and likely experiences delayed implantation during pregnancy [6]; however, detailed profiles of this species' estrous cycle and embryonic development only became possible following the establishment of a captive population, routine monitoring of ovarian steroids excreted in urine [5, 8] and ultrasonography [9]. Captive studies have determined that physiological changes preceding estrus last 1–2 weeks, peak receptive behaviors and natural breeding occur for 1–3 days around ovulation [10–13] and an extended period of luteal activity follows ovulation [5, 14]. Furthermore, progestagen metabolite concentrations increase after ovulation in two distinct phases, regardless of whether breeding or conception occurs, and the pattern and duration of the increase are indistinguishable between pregnant and non-pregnant females–a phenomenon known as pseudopregnancy [5, 14]. The primary rise in progestagens is highly variable in length, lasting 1–4 months, and is followed by a larger, more consistent secondary increase that lasts 45–55 days, with pregnant females giving birth 85–181 days after breeding, as progestagens return to baseline [15–18]. The exceptional variability in the duration of the primary progestagen rise is related to embryonic diapause [18]. The mechanisms controlling the reactivation of corpus luteal function and embryonic development of implantation in the giant panda are unknown. Hormonal data, ultrasound analysis, and the size of altricial young support the hypothesis that embryo implantation occurs sometime between the onset of the secondary rise in progesterone and the peak in progesterone, allowing for less than 40 days of post-implantation fetal growth [6, 16, 18–20].

Confirmation of pregnancy status and prediction of parturition timing for this species with wildly variable gestation lengths and vulnerable altricial young is important for *ex situ* conservation efforts. But in addition to the similarities in progestagen profiles between pregnant and pseudopregnant giant pandas, behavioral and physiological changes are remarkably similar. In both physiological states, females demonstrate maternal behavior (i.e., denning and cradling objects), decreased appetite, mammary gland growth, and changes in vulva morphology [5]. Urinary or fecal concentrations of other hormones, including estrogens, relaxin and glucocorticoids, also have proven to be ineffective indicators of pregnancy [16, 20, 21]. Ultrasonography is widely used in captive giant panda breeding programs to monitor uterine changes, detect pregnancy, and predict parturition date [9, 22]. However, pregnancy detection by ultrasonography is limited to 2–3 weeks prior to birth, requires a trained and cooperative panda during the late luteal phase when females often become unresponsive to behavioral training [9] and even when ultrasonography is performed during critical times, fetuses have gone undetected in multiple successful pregnancies (J. Brown and R. Snyder, per communication). An alternative imaging system, thermography, has been used to distinguish pregnancy from pseudopregnancy and detect fetal heat signatures several days in advance of ultrasound visualization [23]. Although this a promising diagnostic technique it has not been widely adopted—likely because it still requires a trained and cooperative bear as well as expensive equipment not commonly on hand at captive facilities. The limitations of imaging techniques further demonstrate the need for a biochemical test for pregnancy in bears.

Recent research of urinary levels of ceruloplasmin, an acute phase protein associated with inflammation, showed that ceruloplasmin increases in the urine pregnant, but not pseudopregnant, giant pandas as early as 1 week after conception [7]. Ceruloplasmin decreases to baseline the last 3 to 4 weeks of pregnancy; coincident to the timing for first visualization of the embryo by ultrasound [7, 20]. Kersey [20] hypothesized that nidation may occur around this time. The use of ceruloplasmin as a reliable biomarker of pregnancy; however, may be limited because the protein is relatively unstable and daily variation in concentrations requires intensive serial sampling and real-time monitoring throughout the luteal phase [7]. Given these constraints, the development of additional biomarkers of pregnancy is needed.

One such potential candidate is prostaglandin F_2α_ (PGF_2α_). This hormone is produced in multiple tissues, including the uterus and fetus, and is involved in corpus luteum function, implantation, initiation of parturition, and resumption of post-partum ovarian cycling [24–26]. Circulating PGF_2α_ is quickly metabolized to 13,14-dihydro-15-keto-PGF2α (PGFM) in the lungs and excreted from the body by the renal system [27]. This inert metabolite is relatively stable, and concentrations in serum are correlated with PGF_2α_ activity in several species, including the human [28], ewe (*Ovis aries*) [29], and buffalo (*Babalus babalis*) [30]. Importantly, PGFM also is a well-established marker of pregnancy in two species that experience pseudopregnancy, the domestic dog (*Canis lupus familiaris*) [31–33] and cat (*Felis catus*) [34]. In both species, serum PGFM increases in pregnant, but not pseudopregnant females, starting around 30 days before parturition. Identification of these pregnancy-specific increases has led to the recent exploration of PGFM as a biomarker of pregnancy in non-domesticated species.

To be a useful biomarker of pregnancy for intractable wildlife, PGFM must be quantifiable through non-invasive monitoring using urine or feces. This application was first explored in the Iberian lynx (*Lynx pardinus*), a critically endangered felid endemic to southwestern Spain [1], in which both urinary and fecal PGFM concentrations were found to consistently distinguish pregnancy from pseudopregnancy [35]. However, subsequent research revealed that fecal PGFM patterns vary among felids, and are not predictive of pregnancy in every species [36]. For felids where PGFM concentrations can be used to detect pregnancy, concentrations increase gradually, starting 2–6 weeks before parturition, and return to baseline 1–4 days after birth [36, 37]. Very little is known about the role of prostaglandins in pregnancy of other carnivores, such as ursids. A preliminary study comparing PGFM values from a single giant panda during a pseudopregnancy (2002) and a pregnancy (2005) found that fecal PGFM did not correlate with reproductive status [38]. However, monitoring PGFM in the excretory route of urine has not been explored and may be more suitable than feces, as has been the case for pregnancy specific hormones in other species, such as relaxin [39] and human chorionic gonadotrophin (hCG) [40]. Given the giant panda’s unique reproductive physiology, knowledge about PGFM patterns in this species may provide novel insights into mammalian reproduction, as well as a useful biomarker of pregnancy for this species. The main objectives of our study were to: 1) characterize urinary PGFM profiles in the female giant panda; and 2) determine if PGFM can be used to accurately determine pregnancy status and predict timing of parturition.

## Materials and methods

### Animals and sample collection

Female giant pandas (n = 4), identified by their international studbook numbers (SB) [3], were housed at the Memphis Zoo (SB507), San Diego Zoo (SB371), Smithsonian’s National Zoological Park (SB473), and Zoo Atlanta (SB452). Male giant pandas (n = 1 per institution) were housed near females. The giant pandas were managed utilizing best practices for animal husbandry and management guidelines set forth by zoological institutions with previous giant panda experience, the American Zoo Association (AZA), Chinese husbandry standards, and approved by U.S. Fish and Wildlife Service (USFWS). Fresh water was always available, and all animals were fed a diet consisting of 75–90% bamboo supplemented with various items depending on the institution; including, high fiber biscuits, fruits, juice, diluted honey water, sugar cane, carrots and yams. Male and female giant pandas were housed in close proximity to allow for auditory, olfactory, and visual contact between pairs. Direct physical contact was limited outside of estrus. During years that females were bred, pairs were allowed physical contact during behavioral estrus, when females demonstrated receptive behaviors.

Urine was routinely collected in a non-invasive manor that does not disturb the animals. These samples were collected for health monitoring as part of veterinary and husbandry management. Freshly voided urine samples (2–10 ml) were collected 3 to 5 times a week and stored frozen (-20°C) until analysis. Samples were non-invasively collected from the floor as part of routine husbandry, with care to avoid contamination from fecal or water. Dates of physiological changes indicative of estrus, copulation and/or AI, and parturition were recorded to establish physiologic relationship with hormones. The National Zoo, San Diego Zoo, Zoo Atlanta and Memphis Zoo collected samples for giant panda veterinary/husbandry management over multiple years and shared aliquots of frozen stored samples for the current study; no additional samples were collected for the current study. Samples were collected from 1997 through 2012, dependent on when giant pandas arrived at respective institutions and estrus occurred. Aliquots of urine samples were shipped frozen to the Memphis Zoo and stored (-20°C) until analysis.

We analyzed PGFM concentrations in samples collected throughout six successful pregnancies (n = 3 females, 2 pregnancies each) and four pseudopregnancies (n = 1 per female) (Table 1). One pregnancy resulted from natural breeding, while the remaining pregnancies resulted from artificial insemination (AI). For the four pseudopregnancies analyzed, the females were not naturally or artificially bred to ensure that females were not pregnant. This stipulation eliminated the inclusion of years a female may have experienced pregnancy loss after breeding. Urinary estrogen and progesterone metabolites were measured at the perspective institution the year of collection as part of routine reproductive management. We used peri-ovulatory changes in urinary estrogen and progesterone metabolite concentrations to align the 10 PGFM profiles from ovulation and examined the post-ovulatory relationship of progesterone and PGFM during pregnancy and psuedopregnant diestrus. Pregnancy and pseudopregnancy treatment groups for the current study were based on nonmanipulated physiological events. The study was carried out in accordance to Memphis Zoo Animal Care and Use Committee policies; studies conducted with urine are considered noninvasive and do not require approval for use.

**Table 1:** Giant panda reproductive cycles monitored for urinary PGFM concentrations.

Studbook #	Zoo	Peak Estrus	Breeding/AI*	Luteal Phase (days)
371	San Diego	4/25/97	No	106
		4/9/99	Yes	133
		3/22/03	Yes	150
452	Atlanta	3/30/03	No	124
		3/28/06	Yes	161
		6/15/10	Yes	140
473	National	4/26/02	No	162
		3/10/05	Yes	121
		4/29/12	Yes	140
507	Memphis	9/26/05	No	122

*All breeding/artificial insemination (AI) attempts resulted in pregnancy and parturition.

### Urinary PGFM analysis

Concentrations of immunoreactive PGFM in urine samples were measured using a DetectX^®^ PGFM enzyme immunoassay (EIA) kit (Arbor Assays, Ann Arbor, Michigan; K022). The manufacturer-specified limit of detection is 46.2 pg/ml, and cross-reactivity of the PGFM antiserum to PGFM is 100% and 1.5% for PGEM. The PGFM antibody is not cross-reactive with any other eicosanoid tested including PGF_2α_, PGE_2,_ tetranor-PGFM, tetranor-PGEM, 11β-PGF_2α_, PGF_2β,_ and PGAM. The assay was performed following the manufacturer’s instructions, except that incubation was extended to 1.5 hours, increasing total binding by ~10%. Prior to analysis, urine samples were diluted in assay buffer to ensure 20–80% antibody binding within the dynamic range (50–3200 pg/ml) of the assay. This corresponded to 5- to 20-fold and 100- to 800-fold dilution factors during baseline and elevated periods, respectively. Optical density was read at 450 nm and values were converted to hormone metabolite concentrations using MRX Revelation Software (Thermo Scientific; Rochester, NY). Urine samples were analyzed for creatinine (Cr) content [41, 42], and final urinary hormone concentrations were reported as ng/mg Cr.

### PGFM assay validation

The PGFM assay was validated for giant panda urine by testing parallelism, accuracy, precision, and stability. To examine parallelism, standards and samples were serially-diluted, and resulting curves were plotted as relative concentration versus percent bound antibody and non-linear regression was performed. Assay accuracy was assessed by determining the percent recovery of known amounts of PGFM (1600–100 ng/ml) added to urine samples containing low endogenous PGFM. Hormone recovery was assessed using linear regression of observed to expected hormone concentrations. The intra-and inter-assay coefficients of variation were determined by use of two internal controls at high (800 pg/ml) and low (100 pg/ml) concentrations run in duplicate on each plate. To evaluate PGFM stability, urine samples from females SB507 (n = 2), SB452 (n = 2), and SB473 (n = 1) ranging in concentration (989, 536, 326, 288 and 126 pg/ml), were subjected to six freeze-thaw cycles, with subsamples taken for analysis after each cycle.

### Estrogen and progestagen analysis

Concentrations of immunoreactive urinary estrogen and progestagen metabolites were measured within 12 months of sample collection using previously validated EIAs [41] for three of the female giant pandas (SB452, SB473, and SB507) or a radioimmunoassay (RIA) system [43] for the remaining female giant panda (SB371). Antibodies and horseradish peroxidase (HRP) conjugates for all assays were obtained from C. Munro (University of California, Davis, CA). These analyses were conducted at the pandas’ respective institutions, except for SB452 samples, which were also analyzed at the Smithsonian Conservation Biology Institute. The progesterone EIA used a progesterone standard, antibody (CL425) and HRP conjugate. The pregnanediol-3-glucuronide (PdG) RIA used a PdG standard with PdG antibody (P-26) and conjugate. Antibody cross-reactivity for the PdG and progesterone antisera has been previously described [43, 44]. Both EIA and RIA assays for estrogen metabolites used the estrone-3-glucuronide antibody (E1G; R522–2) and estrone-3CMO-HRP conjugate. For standards, Memphis Zoo used estrone 3(β–D–glucuronide) (7.85–4,000 pg/ml), while the remaining institutions used 1, 3, 5(10)-estratrien-17-one 3-sulfate (39–10,000 pg/ml). The E1G antisera crossreactivity is 100% with estrone-3-glucuronide, 66.6% with estrone-3-sulfate, and 238% with estrone [43]. Final hormone concentrations are reported as ng/mg Cr. All reagents and standards were obtained from Sigma Aldrich (St. Louis, MO) unless otherwise noted.

### Statistical analysis

Descriptive statistics and iterative analyses were conducted using Microsoft Excel 2010 (Microsoft Corporation, Redmond, WA). All remaining analyses were conducted using Sigma Plot 12.0 (Systat Software Inc, San Jose, California) and data were considered significant at *P* < 0.05. Hormone data were log-transformed for analysis, and approximate normality was confirmed using the Shapiro–Wilks test.

Baseline values for each hormone profile were determined by an iterative process as described previously [8]. Briefly, baseline was determined by removing values greater than two standard deviations (SD) above the mean from the data set; mean values were recalculated and the process repeated until no values exceeded two SD above the mean. The mean of the remaining values was determined to be baseline. For both PGFM and progestagens, the end of the basal period was defined as the point where values exceeded two SD above baseline for two consecutive days. Similarly, for progestagen profiles, the onset of the secondary rise was defined as the point where values exceeded the primary phase baseline by at least two SD for two consecutive days. The secondary rise ended with either parturition in pregnant females or return to baseline progestagen concentrations in pseudopregnant females. For all hormone profiles, the day estrogen metabolites precipitously decreased from peak levels and progestogens began to increase was designated Day 0, representing peri-ovulation and start of the luteal phase [10, 11, 45]. All estrogen and progestagen profiles except for the 2012 profile for SB473 were reported previously [7, 14]. Differences in hormone values between pregnant and pseudopregnant PGFM profiles were determined using a Student’s *t*-test, while differences among time intervals were assessed by one-way analysis of variance (ANOVA) (*F*-statistic) with Holm-Sidak post-hoc comparisons. Percent PGFM recovery after each freeze-thaw cycle was compared using repeated measures ANOVA (*F*-statistic). Urinary estrogen was not monitored throughout the luteal phase for all the profiles; therefore only the relationship of urinary progestogens and PGFM during pregnancy and pseudopregnancy was explored.

## Results

### PGFM assay validation

**Fig 1:**
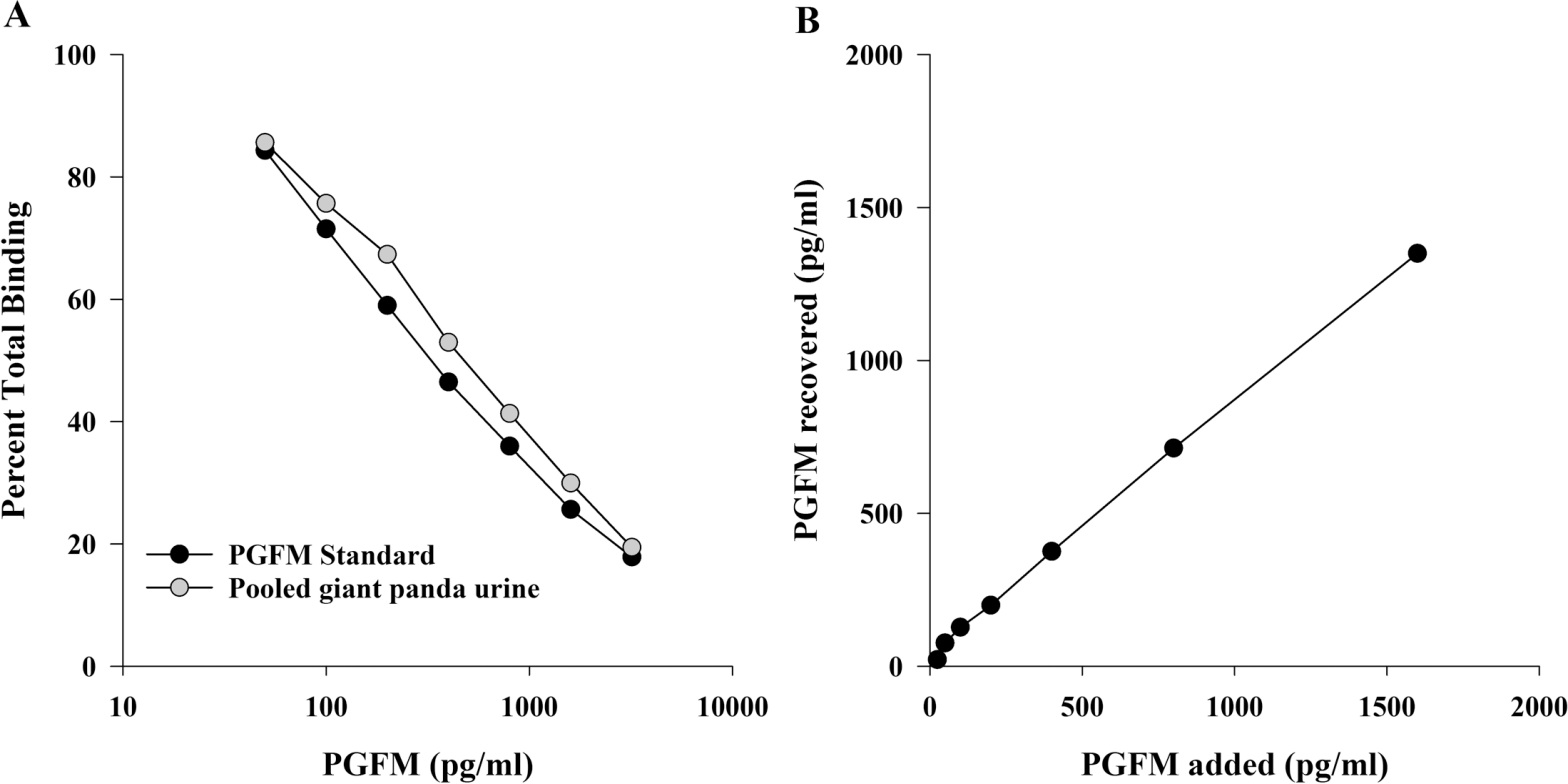
PGFM assay validation. (A) Parallelism: Diluted giant panda urine and PGFM standard demonstrate parallel displacement (R^2^ = 0.999, *P* < 0.001). (B) Recovery: Validation that components of panda urine did not interfere with recovery of exogenous hormone spiked into urine (R^2^ = 0.999, *P* < 0.001).

Serially-diluted urine samples demonstrated displacement curves parallel (*R*^2^ = 0.999, *P* < 0.001) to those obtained for the PGFM standards (Fig 1A). The mean percentage recovery of spiked urine samples was 102 ± 7% (y = 0.83x + 31.94, *R*^2^ = 0.999, *P* < 0.001, Fig 1B). Inter-assay coefficients of variation (CV) of high (800 pg/ml) and low (100 pg/ml) PGFM controls run on each plate were 13% and 16%, respectively (n = 59), and intra-assay CV was <10%. Percentage PGFM recovered in frozen-thawed samples (96.9 ± 3.4%) were not influenced by original sample concentration or the number of consecutive freeze-thaw cycles (*P* = 0.557). The parallelism, spike-recovery, and freeze-thaw tests validated the use of PGFM assay for giant pandas and that concentrations from banked samples could be compared.

### PGFM profiles in pregnant versus pseudopregnant females

**Fig 2:**
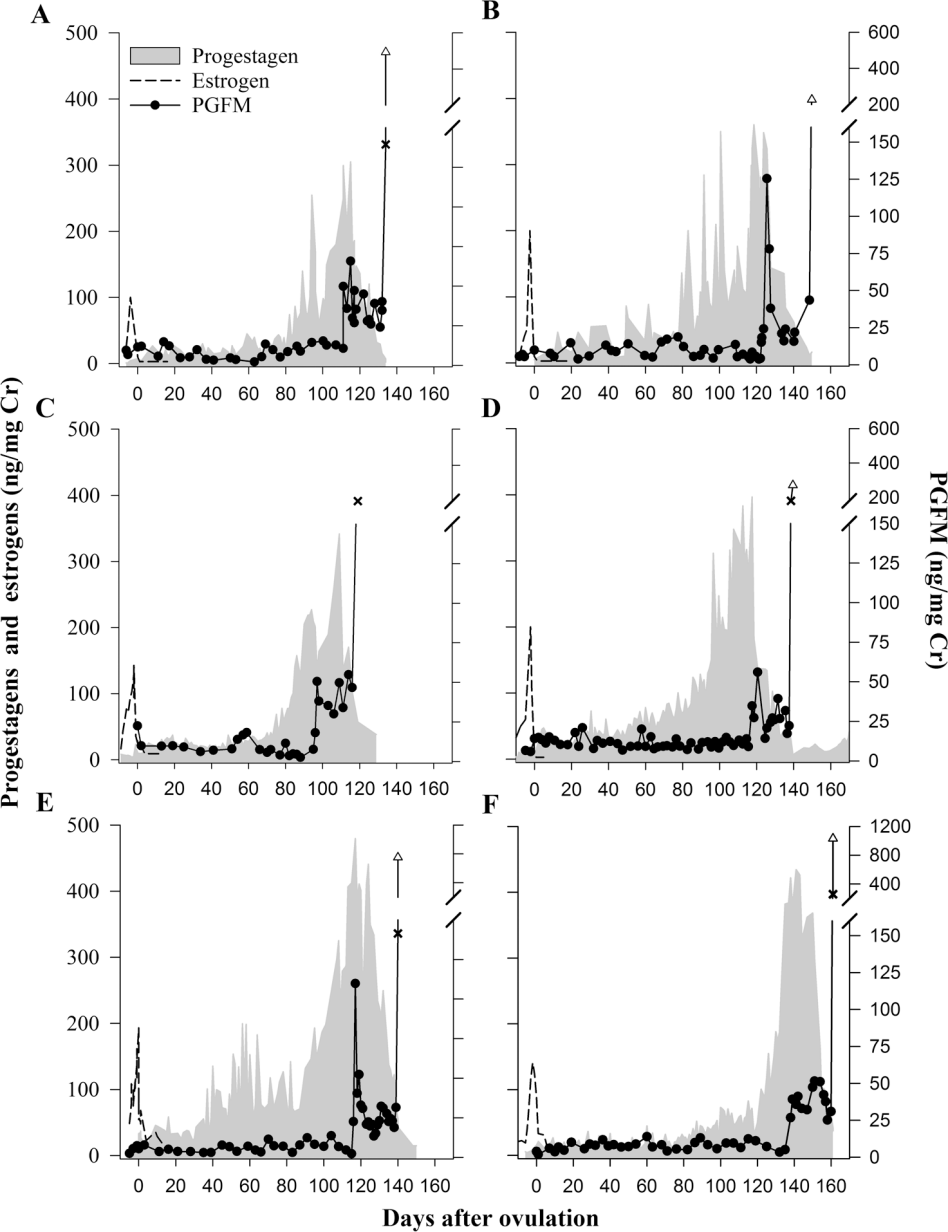
Urinary hormone profiles during six representative giant panda pregnancies. Urinary estrogen, progestagen, and PGFM concentrations relative to ovulation from four giant pandas, representing six gestations: SB371 in 1999 (A) and 2003 (B), SB473 in 2005 (C), SB452 in 2010 (D), SB473 in 2012 (E) and SB452 in 2006 (F). Black circles represent urinary PGFM concentrations and samples collected 12–24 hours (×) and 5–10 hours (∆) before parturition are notated. Note the difference in scale of secondary x-axis for graphs A–D versus E–F.

**Fig 3:**
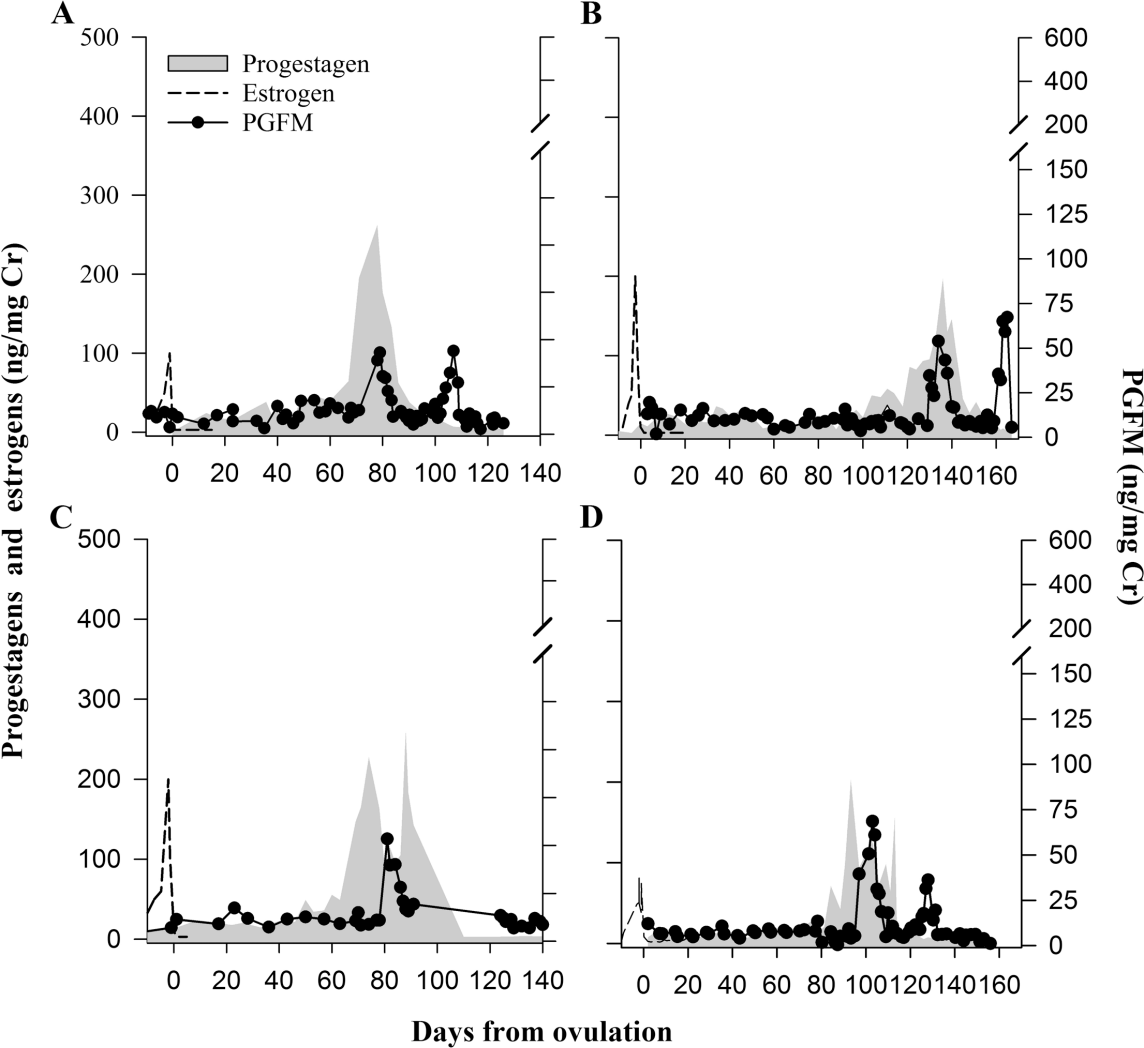
Urinary hormone profiles during four giant panda pseudopregnancy when no breeding occurred. Urinary estrogen, progestagen, and PGFM relative to ovulation in four non-bred female giant pandas: SB371 in 1997 (A), SB473 in 2002 (B), SB452 in 2003 (C), SB507 in 2005 (D). Axis scales are consistent with Fig 2A–2D.

**Fig 4:**
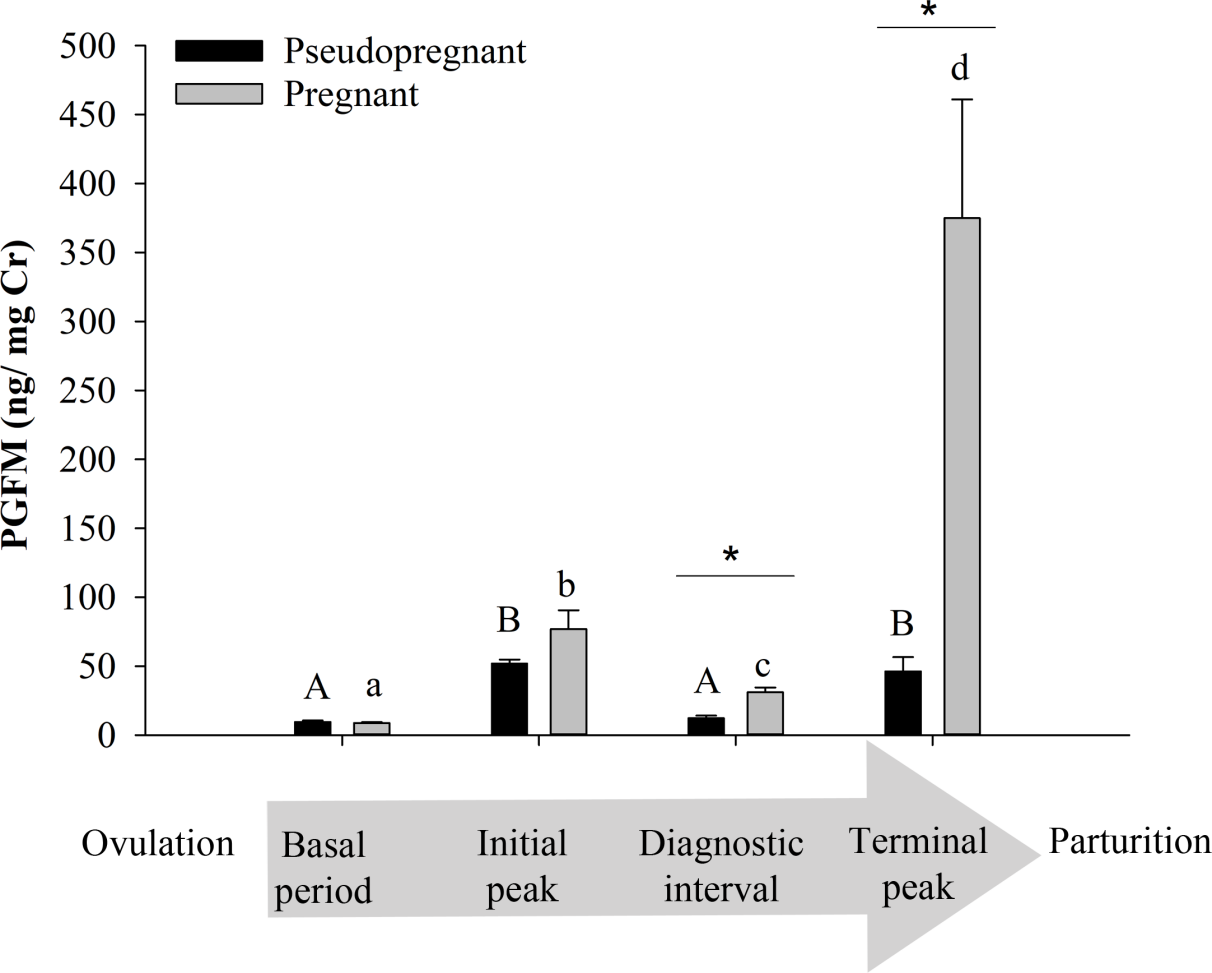
Mean PGFM concentrations during giant panda pregnancy versus pseudopregnancy. Urinary PGFM concentrations (mean ± SE) relative to time period in four pseudopregnancies and six pregnancies (n = 4 females total). Periods are positioned in chronological order along the x-axis, as indicated by the shaded arrow. Within each reproductive status group, differences (*P* < 0.05) among time periods are represented by different superscripts. Within each time period, differences (*P* < 0.05) between pregnant and pseudopregnant groups are indicated by an asterisk (*).

Urinary PGFM was detectable before ovulation and throughout all pregnancies (Fig 2) and pseudopregnancies (Fig 3). For all profiles examined, PGFM concentrations remained at baseline for 11–19 weeks after ovulation, ranging from 3.60 to 13.50 ng/mg Cr (Mean 9.26 ± 1.60 ng/mg Cr). Baseline PGFM concentrations did not differ (*P* = 0.494) between pregnant and pseudopregnant females (Fig 4). In both groups, a PGFM surge began 2 to 5 weeks after the start of secondary rise of progestagens, at which time PGFM concentrations approximately tripled within 24 hours. This increase occurred later (*P* < 0.028) in pregnant females compared to pseudopregnant females (31.7 ± 3.1 days versus 21.0 ± 2.8 days after the start of the secondary rise in progestogens, respectively). Likewise, the interval between the initial increase in PGFM to parturition was shorter (24.0 ± 1.0 days, *P* < 0.001) than to the end of the luteal phase of pseudopregnant females (33.0 ± 0.5 days). In both groups, this initial PGFM surge lasted 10–14 days and peaked at values 4-7-fold higher (*P* < 0.001) than basal concentrations. Peak concentrations were not different (*P* = 0.185) between pregnant and pseudopregnant females (76.83 ± 13.66 vs. 51.83 ± 3.00 ng/mg Cr, respectively; Fig 4). Following this initial surge, PGFM concentrations returned to baseline in pseudopregnant females (12.28 ± 1.74 ng/mg Cr), but remained elevated above baseline (*P* < 0.001) in pregnant females (31.04 ± 3.42 ng/mg Cr, *P* < 0.001) and concentrations were significantly different (*P* = 0.003) between the two groups (Fig 4).

Pregnant females then experienced a second, significantly larger, PGFM surge starting 12 to 24 hours prior to birth, with the peak coinciding as progestagen concentrations declined to baseline (Fig 2). Pseudopregnant giant pandas also exhibited a second increase in urinary PFGM at the end of the luteal phase, but peaks generally occurred a few days after the progestagens returned to baseline, and at much lower amplitude (Fig 3). A second PGFM increase could not be confirmed in one pseudopregnancy (SB452 in 2003) because samples were not collected during that time period. For five of the six pregnancies, two samples were collected during the last 24-hours prior to birth and demonstrated that concentrations exponentially increased (*P* = 0.002) throughout the day of birth (Fig 2). Peak PGFM concentrations during this second surge were nearly 8-fold greater (*P* = 0.035) in pregnant versus pseudopregnant females (374.97 ± 86.09 and 46.23 ± 10.23 ng/mg Cr, respectively; Fig 4).

## Discussion

As a result of this study we were able to determine that prostaglandins play a role in regulating corpus luteum activity and pregnancy in the giant panda; and the metabolites of which, PGFM, can be used as a biomarker of pregnancy status. Moreover, we identified distinct differences in urinary PGFM excretory patterns between pregnant and pseudopregnant giant pandas, along with biomarker indicators that allow for accurate timing of parturition in this species. In general, pregnant giant pandas exhibited a tri-phasic urinary profile of PGFM, which included an initial rise, followed by a period of moderately elevated PGFM (which we term the ‘diagnostic interval’), and finally a larger, terminal spike near the time of parturition. The giant panda appears to be different in that pseudopregnant females also experienced an increase in PGFM, which contrasts with patterns observed in felids and canids, where significant PGF_2α_ and PGFM elevations during the luteal phase are observed only in pregnant animals promoting active luetolysis and parturition [31, 34, 35]. Despite this, key differences in timing and magnitude of urinary PGFM changes exist, which suggests this test can be used reliably to determine pregnancy status and predict parturition. The timing of the initial PGFM rise after the start of the secondary progestagen phase started later for pregnant females and occurred consistently 24 +/- 1 days prior to parturition. Furthermore, the timing and magnitude of the terminal PGFM peak provides an unequivocal signal of impending parturition.

The tri-phasic pattern of PGFM concentration observed in the giant panda appears to be unique among mammals. To our knowledge, this is the only species that exhibits an initial spike in PGFM several weeks before parturition, followed by a period of relatively low, albeit still elevated, concentrations that last until the peri-parturient surge. By contrast, other mammals demonstrate either a single, peri-parturient surge or a gradual increase throughout the last 10–30 days of gestation that peaks near the time of parturition [30, 31, 35, 46–48]. We also found that the initial PGFM increase in pregnant giant pandas occurs around the time that ceruloplasmin returns to baseline during the secondary rise in progestogens and 3–5 days before the embryo can be first visualized by ultrasound [7, 9, 18, 20]. The underlying relationship of this unique PGFM pattern to its physiological mechanism are not understood in the giant panda, but our findings lend further evidence that hormonal and physiological changes correlated to implantation and fetal growth occur less than 30 days before parturition. In many species that exhibit delayed implantation (i.e., spotted skunk, European badger, mink, and ferret) an increase in progesterone alone is not sufficient to induce implantation [49], rather, other luteal, uterine and/or neuroendocrine factors act in conjunction with progesterone to initiate implantation [49, 50]. The intriguing possibility that maternal or fetal derived PGF_2α_ is part of a hormonal cascade regulating implantation, early fetal development, luteal regression, and parturition in the giant panda is a priority for further investigation.

The giant panda is unusual in that both pregnant and pseudopregnant females experience a PGFM increase after the secondary rise in progestagens. This pattern contrasts with the lack of a PGFM increase during pseudopregnancy in the dog [31], cat [34], and some non-domestic felids, including the cheetah (*Acinonyx jubatus*), Iberian lynx (*Lynx pardinus*), sand cat (*Felis margarita*), and puma (*Puma concolor*) [35, 48]. Although the timing, duration and concentration of PGFM is different between pregnant and pseudopregnant giant pandas, the fact that the profiles are similar between the two physiological states make teasing out the exact mechanism of PGF_2α_ in various reproductive functions challenging. However, these observations suggest that corpus luteum function in the pseudopregnant giant panda may be actively regulated by PGF_2α_, in contrast to the dog and cat, where it has a fixed lifespan and luteolytic PGF_2α_ is not observed during pseudopregnancy [26, 51]. The physiological reason for this species difference is unclear, but may be related to embryonic diapause which occurs in the giant panda [18] but is absent in canids and felids. If PGF_2α_ regulates both embryo implantation and luteal regression in the giant panda, its presence would be expected around these time frames. This hypothesis is consistent with the timeline of PGFM secretion observed in the present study.

**Fig 5:**
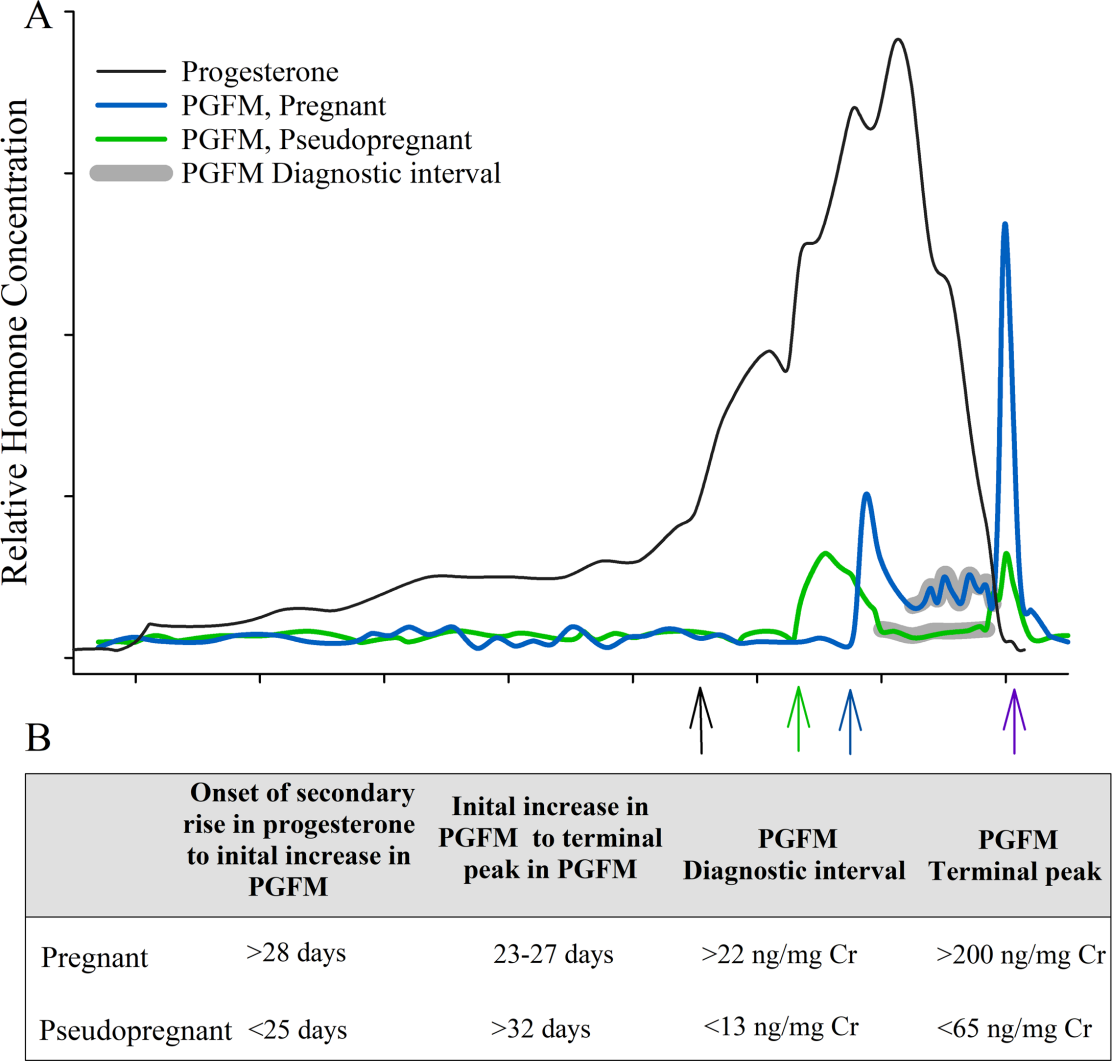
Schematic summary of urinary PGFM and the relationship to progestogen levels during giant panda pregnancy and pseudopregnancy. (A) Schematic of hormone profiles in parturient versus pseudopregnant pandas and (B) diagnostic features of PGFM profiles for determining pregnancy status. These diagnostic features are emphasized in the schematic, and include an initial PGFM increase (blue and green arrows) that occurs after the secondary P4 rise (black arrow), followed by a terminal PGFM peak (blue arrow) that occurs within 12 h of parturition in pregnant animals. Between the two PGFM peaks is a diagnostic interval (shaded gray areas), during which concentrations of the metabolite differ in parturient and pseudopregnant females. (Values are one standard deviation from mean).

Despite the overall similarities in PGFM profiles, we observed distinct differences in urinary concentrations of this metabolite between pregnant and pseudopregnant giant pandas. A schematic representing the distinctive PGFM patterns during pregnancy and psuedopregnancy is depicted in Fig 5. Pregnancy status could be determined by the timing of the initial PGFM peak, and by the concentrations of the terminal peak and the diagnostic interval. During pregnancy, the initial PGFM increase occurred 28–36 days into the secondary progestagen rise and 23–27 days prior to birth. In contrast, non-bred psuedopregnant females, had an initial PGFM rise within 14–25 days into the secondary progestagen rise and more than 32 days before the end of the luteal phase. Definitive confirmation of the onset of the secondary progestagen rise can be difficult in real time, complicating PGFM-based pregnancy determination. Yet, PGFM concentrations following this initial peak can provide confirmation of suspected pregnancy status. Thus, an additional factor to consider is the mean inter-spike (diagnostic interval) PGFM concentration. Each pregnant female exhibited a mean PGFM concentration greater than 22 ng/mg Cr during the diagnostic interval, which was significantly higher than non-bred females (<13 ng/mg Cr) and baseline concentrations. Importantly, we observed daily variation in PGFM concentrations during the diagnostic interval among all animals (14–51 ng/mg Cr in parturient, 5–19 ng/mg Cr in non-bred), making it necessary to focus on *moving averages* versus instantaneous values during this period. Final confirmation of pregnancy was the dramatic second surge in PGFM that began within 24-hours of birth, peaked above 200 ng/mg Cr, and was >3-fold higher than the highest terminal peak observed in non-bred females (65 ng/mg Cr). This exponential peri-parturient increase in PGFM is consistent with observations in the human [28], rat [52], rabbit [53], domestic dog and cat [33, 34], and various livestock species [46, 47, 53].

While other methods (e.g., ultrasound) can be effective at diagnosing pregnancy in the giant panda, urinary PGFM monitoring represents the only quantitative predictor for the timeframe of parturition. The consistent timing of the initial PGFM peak, duration and concentration of the diagnostic interval PGFM allow for an accurate prediction of birth date several weeks in advance. Thus, urinary PGFM monitoring represents a highly valuable tool for keepers, managers, and veterinarians that are overseeing *ex situ* giant panda conservation management and husbandry. By using information from the PGFM monitoring these zoological professionals can better prepare their schedules, staff, supplies, and facilities for a pending birth. For example, the onset of the peri-parturient PGFM spike provides further confirmation that birth will occur within 12–24 hours and instructs keepers that the female should potentially be confined inside and within the birthing area/den. For managers of giant panda husbandry within captive facilities, precise prediction of parturition timing is critical because it facilitates staff scheduling for round-the-clock, 24-hour animal care support. Such support is key to ensuring cub survival because giant pandas deliver altricial infants with a high potential of mortality within hours of parturition due to rejection, neglect, or insufficient lactation [6, 54]. Furthermore, our study provides evidence that PGF_2α_ increases around the time of successful implantation and parturition, suggesting that further exploration of PGF_2α_ and other factors around these PGFM spikes may reveal warning signals of pregnancy loss in this species. Pregnancy loss after implantation has been documented in captive giant pandas [7] and the detection of signals correlated to pregnancy loss may help managers mitigate issues and improve reproductive success.

Our study revealed a complex pattern of PGFM secretion that has not been observed in other mammals. The timing of this pattern suggests that PGF_2α_ may play a key role in luteal activity, early fetal development, and parturition—an intriguing possibility that warrants further investigation. In addition to determining that urinary PGFM monitoring can provide a non-invasive method of diagnosing pregnancy and predicting parturition date, we have documented evidence of active luteolytic factors during both pregnancy and pseudopregnancy, and pinpointed key time periods where further study of the endocrine milieu involving PGF_2α_ may reveal further insights into predictors of pregnancy success and loss in the giant panda. Novel tools for detecting pregnancy are urgently needed for the giant panda given the reliance on *ex situ* populations as a hedge against extinction and for reintroduction efforts. Our study provides a promising tool for pregnancy detection and highlights some of the mysteries that remain surrounding the reproductive physiology of this charismatic ursid.

## Supporting information

S1 File Personal communication letter, Dr. Janine Brown. (PDF)

S2 File Personal communication letter, Rebecca Snyder. (PDF)

S3 File Urinary PGFM giant panda data. (XLSX)

## References

[1] The IUCN Red List of Threatened Species. In. http://www.iucnredlist.org Version 2016–2; 2016: Downloaded on 04 September 2016.

[2] Swaisgood R, Wang D, Wei F. Ailuropoda melanoleuca. In: The IUCN Red List of Threatened Species 2016. e.T712A45033386: Downloaded on 05 October 2016; 2016.

[3] Xie Z. The 2015 International studbook for giant panda (Ailuropoda melanoleuca) Beijing P. R. China: Chinese Association of Zoological Gardens; 2015.

[4] Czekala N, McGeehan L, Steinman K, Xuebing L, Gual-Sil F. Endocrine monitoring and its application to the management of the giant panda. Zoo Biology 2003; 22:389–400.

[5] Steinman KJ, Monfort SL, McGeehan L, Kersey DC, Gual-Sil F, Snyder RJ, et al Endocrinology of the giant panda and application of hormone technology to species management In: Wildt DE, Zhang AJ, Zhang H, Janssen DL, Ellis S (eds.), Giant Pandas: Biology, Veterinary Medicine, and Management. United Kingdom, Cambridge: Cambridge University Press; 2006: 198–230.

[6] Schaller GB, Hu J, Pan W, Zhu J. The Giant Pandas of Wolong Chicago, IL, USA: The University of Chicago Press; 1985.

[7] Willis EL, Kersey DC, Durrant BS, Kouba AJ. The acute phase protein ceruloplasmin as a non-invasive marker of pseudopregnancy, pregnancy, and pregnancy loss in the giant panda. Plos One 2011; 6:1–11. 10.1371/journal.pone.0021159 PMC3135589 21765892

[8] Monfort S, Dahl K, Czekala N, Stevens L, Bush M, Wildt D. Monitoring ovarian function and pregnancy in the giant panda (Ailuropoda melanoleuca) by evaluating urinary bioactive FSH and steroid metabolites. Journal of Reproduction and Fertility 1989; 85:203–212. 2492603 10.1530/jrf.0.0850203

[9] Sutherland-Smith M, Morris PJ, Silverman S. Pregnancy detection and fetal monitoring via ultrasound in a giant panda (Ailuropoda melanoleuca). Zoo Biology 2004; 23:449–461.

[10] Durrant B, Olson M, Amodeo D, Anderson A, Russ L, Campos-Morales R, et al Vaginal cytology and vulvar swelling as indicators of impending estrus and ovulation in the giant panda (*Ailuropoda melanoleuca)*. Zoo Biology 2003; 22:313–321.

[11] Kersey DC, Wildt DE, Brown JL, Snyder RJ, Huang Y, Monfort SL. Endocrine milieu of perioestrus in the giant panda (Ailuropoda melanoleuca), as determined by non-invasive hormone measures. Reproduction, Fertility and Development 2010; 22:901–912. 10.1071/RD09178 20591324

[12] Czekala NM, Lindburg DG, Durrant BS, Swaisgood RR, Tingmei H. The Estrogen profile, vaginal cytology, and behavior of a giant panda female during estrus In: International symposium on the Environmental Protection and City Development of 21st century Chendgu, China; 1997.

[13] Durrant B, Czekala N, Olson M, Anderson A, Amodeo D, Campos-Morales R, et al Papanicolaou staining of exfoliated vaginal epithelial cells facilitates the prediction of ovulation in the giant panda. Theriogenology 2002; 57:1855–1864. 12041689 10.1016/s0093-691x(02)00678-7

[14] Kersey DC, Wildt DE, Brown JL, Snyder RJ, Huang Y, Monfort SL. Unique biphasic progestagen profile in parturient and non-parturient giant pandas (Ailuropoda melanoleuca) as determined by faecal hormone monitoring. Reproduction 2010; 140:183–193. doi: 10.1530/REP-10-0003 20406954 10.1530/REP-10-0003

[15] Kersey DC, Wildt DE, Brown JL, Snyder RJ, Huang Y, Monfort SL. Rising fecal glucocorticoid concentrations track reproductive activity in the female giant panda (Ailuropoda melanoleuca). Gen Comp Endocrinol 2011; 173:364–370. doi: 10.1016/j.ygcen.2011.06.013 21726558 10.1016/j.ygcen.2011.06.013

[16] Hodges JK, Bevan DJ, Celma M, Hearn JP, Jones DM, Kleiman DG, et al Aspects of the reproductive endocrinology of the female Giant panda (Ailuropoda metanoleaca) in captivity with special reference to the detection of ovulation and pregnancy. Journal of Zoology 1984; 203:253–267.

[17] Snyder RJ. Reproduction in Giant Pandas In: Lindburg DG, MacKay P, Ray JC, Zielinski J (eds.), Giant Pandas: Biology and Conservation. Berkeley, CA: University of California Press; 2004: 125–132.

[18] Zhang H, Li D, Wang C, Hull V. Delayed implantation in giant pandas: the first comprehensive empirical evidence. Reproduction 2009; 138:979–986. doi: 10.1530/REP-09-0241 19692498 10.1530/REP-09-0241

[19] Chaudhuri M, Kleiman DG, Wildt DE, Bush M, Frank ES, Thau RB. Urinary steroid concentrations during natural and gonadotrophin-induced oestrus and pregnancy in the giant panda (Ailuropoda melanoleuca). Journal of Reproduction and Fertility 1988; 84:23–28. 3141616 10.1530/jrf.0.0840023

[20] Kersey DC, Aitken-Palmer C, Rivera S, Willis EL, Liu YL, Snyder RJ. The birth of a giant panda: Tracking the biological factors that successfully contribute to conception through to post-natal development. Theriogenology 2016; 85:671–677. doi: 10.1016/j.theriogenology.2015.10.005 26559471 10.1016/j.theriogenology.2015.10.005

[21] Steinetz BG, Brown JL, Roth TL, Czekala N. Relaxin concentrations in serum and urine of endangered species: correlations with physiologic events and use as a marker of pregnancy. Annals of the New York Academy of Sciences 2005; 1041:367–378. doi: 10.1196/annals.1282.057 15956734 10.1196/annals.1282.057

[22] Hildebrandt TB, Brown JL, Goritz F, Ochs A, Morris PJ, Sutherland-Smith M. Ultrasonography to assess and enhance health and reprodcution in the giant panda In: Wildt DE, Zhang AJ, Zhang H, Janssen DL, Ellis S (eds.), Giant Pandas: Biology, Veterinary Medicine, and Management. United Kingdom, Cambridge: Cambridge University Press; 2006: 410–439.

[23] Durrant BS, Ravida N, Spady T, Cheng A. New technologies for the study of carnivore reproduction. Theriogenology 2006; 66:1729–1736. doi: 10.1016/j.theriogenology.2006.02.046 16713619 10.1016/j.theriogenology.2006.02.046

[24] Bergstrom S, Carlson LA, Weeks JR. The Prostaglandins: A family of biologically active lipids. Pharmacological Reviews 1968; 20:1–48. 4873508

[25] Bazer FW. Mediators of maternal recognition of pregnancy in mammals. Experimental Biology and Medicine 1992; 199:373–384. 10.3181/00379727-199-43371a 1549616

[26] McCracken JA, Custer EE, Lamsa JC. Luteolysis: A neuroendocrine-mediated event. Physiological Reviews 1999; 79:263–323. doi: 10.1152/physrev.1999.79.2.263 10221982 10.1152/physrev.1999.79.2.263

[27] Piper PJ, Vane JR, Wyllie JH. Inactivation of prostaglandins by the lungs. Nature 1970; 225:600–604. 4983971 10.1038/225600a0

[28] Mitchell M, Romero R, Edwin S, Trautman M. Prostaglandins and parturition. Reproduction, Fertility and Development 1995; 7:623–632. 10.1071/rd9950623 8606975

[29] Payne JH, Lamming GE. The direct influence of the embryo on uterine PGF_2α_ and PGE_2_ production in sheep. Journal of Reproduction and Fertility 1994; 101:737–741. 7966033 10.1530/jrf.0.1010737

[30] Batra SK, Pahwa GS, Pandey RS. Hormonal milieu around parturition in buffaloes (Bubalus bubalis). Biology of Reproduction 1982; 27:1055–1061. 6961942 10.1095/biolreprod27.5.1055

[31] Luz MR, Bertan CM, Binelli M, Lopes MD. Plasma concentrations of 13,14-dihydro-15-keto prostaglandin F2-alpha (PGFM), progesterone and estradiol in pregnant and nonpregnant diestrus cross-bred bitches. Theriogenology 2006; 66:1436–1441. doi: 10.1016/j.theriogenology.2006.01.036 16464490 10.1016/j.theriogenology.2006.01.036

[32] Baan M, Taverne MAM, De Gier J, Kooistra HS, Kindahl H, Dieleman SJ, et al Hormonal changes in spontaneous and aglepristone-induced parturition in dogs. Theriogenology 2008; 69:399–407. doi: 10.1016/j.theriogenology.2007.10.008 18054071 10.1016/j.theriogenology.2007.10.008

[33] Concannon P, Isaman L, Frank D, Michel F, Currie W. Elevated concentrations of 13, 14-dihydro-15-keto-prostaglandin F-2α in maternal plasma during prepartum luteolysis and parturition in dogs (Canis familiaris). Journal of Reproduction and Fertility 1988; 84:71–77. 3184062 10.1530/jrf.0.0840071

[34] Tsutsui T, Stabenfeldt GH. Biology of ovarian cycles, pregnancy and pseudopregnancy in the domestic cat. Journal of Reproduction Fertility Suppl. 1993; 47:29–35. 8229938

[35] Finkenwirth C, Jewgenow K, Meyer HHD, Vargas A, Dehnhard M. PGFM (13,14-dihydro-15-keto-PGF2α) in pregnant and pseudo-pregnant Iberian lynx: A new noninvasive pregnancy marker for felid species. Theriogenology 2010; 73:530–540. doi: 10.1016/j.theriogenology.2009.10.008 20022361 10.1016/j.theriogenology.2009.10.008

[36] Dehnhard M, Finkenwirth C, Crosier A, Penfold L, Ringleb J, Jewgenow K. Using PGFM (13,14-dihydro-15-keto-prostaglandin F2α) as a non-invasive pregnancy marker for felids. Theriogenology 2012; 77:1088–1099. doi: 10.1016/j.theriogenology.2011.10.011 22192399 10.1016/j.theriogenology.2011.10.011

[37] Dehnhard M, Kumar V, Chandrasekhar M, Jewgenow K, Umapathy G. Non-invasive pregnancy diagnosis in big cats using the PGFM (13,14-dihydro-15-keto-PGF2α) assay. PLoS ONE 2015; 10. 10.1371/journal.pone.0143958 PMC4669140 26633886

[38] Denhhard M, Jewgenow K. Measurement of faecal prostaglandins in felids and three ursid species. Veterinary Medicine Austria 2013; 100:227–237.

[39] de Haas van Dorsser FJ, Lasano S, Steinetz BG. Pregnancy diagnosis in cats using a rapid, bench-top kit to detect relaxin in urine. Reproduction in Domestic Animals 2007; 42. 10.1111/j.1439-0531.2006.00736.x 17214785

[40] Armstrong EG, Ehrlich PH, Birken S, Schlatterer JP, Siris E, Hembree WC, et al Use of a highly sensitive and specific immunoradiometric assay for detection of human chorionic gonadotropin in urine of normal, nonpregnant, and pregnant individuals. Journal of Clinical Endocrinology and Metabolsim 1984; 59:867–874. 10.1210/jcem-59-5-867 6480810

[41] McGeehan L, Li X, Jackintell L, Huang S, Wang A, Czekala NM. Hormonal and behavioral correlates of estrus in captive giant pandas. Zoo Biology 2002; 21:449–466.

[42] Taussky HH, Kurzmann G. A Microcolorimetric determination of creatinine in urine by the Jaffe reacion. Journal of Biological Chemistry 1954; 208:853–862. 13174594

[43] Munro CJ, Stabenfeldt GH, Cragun JR, Addiego LA, Overstreet JW, Lasley BL. Relationship of serum estradiol and progesterone concentrations to the excretion profiles of their major urinary metabolites as measured by enzyme immunoassay and radioimmunoassay. Clin Chem 1991; 37:838–844. 2049848

[44] Graham L, Schwarzenberger F, Mostl E, Galama W, Savage A. A versatile enzyme immunoassay for the determination of progestogens in feces and serum. Zoo Biology 2001; 20:227–236.

[45] Howard JG, Kersey DC, Aitken-Palmer C, Monfort SL, Wildt DE. Capacity of the giant panda to give birth after a single intrauterine insemination using precise ovulation detection. Biology of Reproduction 2008; 78:203.

[46] Senger PL. The luteal phase of the estrous cycle In: Pathways to Pregnancy and Parturition, First Revised ed. Pullman, WA: Current Conceptions, Inc; 1999: 149–165.

[47] Snegovskikh V, Park JS, Norwitz ER. Endocrinology of parturition. Endocrinology and Metabolism Clinics of North America 2006; 35:173–191. doi: 10.1016/j.ecl.2005.09.012 16310648 10.1016/j.ecl.2005.09.012

[48] Dehnhard M, Naidenko SV, Jewgenow K. Comparative metabolism of PGFM (13,14-dihydro-15-keto-PGF2α) in feces of felids. Theriogenology 2014; 81:733–743. doi: 10.1016/j.theriogenology.2013.12.007 24433781 10.1016/j.theriogenology.2013.12.007

[49] Mead RA. Role of the corpus luteum in controlling implantation in mustelid carnivoresa. Annals of the New York Academy of Sciences 1986; 476:25–35. 3541744 10.1111/j.1749-6632.1986.tb20919.x

[50] Lopes F, Desmarais JA, Murphy BD. Embryonic diapause and its regulation Reproduction 2004; 128:669–678. doi: 10.1530/rep.1.00444 15579584 10.1530/rep.1.00444

[51] Jewgenow K, Amelkina O, Painer J, Goritz F, Denhhard M. Life cyle of feline corpora lutea: Histological and intraluteal hormone analysis. Reproduction in Domestic Animals 2012; 47:25–29. doi: 10.1111/rda.12033 23279459 10.1111/rda.12033

[52] Farina M, Ribeiro M, Weissmann C, Estevez A, Billi S, Vercelli C, et al Biosynthesis and catabolism of prostaglandin F_2α_ (PGF_2α_) are controlled by progesterone in the rat uterus during pregnancy. The Journal of Steroid Biochemistry and Molecular Biology 2004; 91:211–218. doi: 10.1016/j.jsbmb.2004.05.001 15336698 10.1016/j.jsbmb.2004.05.001

[53] Thorburn GD, Challis JRG. Endocrine control of parturition. Physiol. Rev. 1979; 59:863–916. doi: 10.1152/physrev.1979.59.4.863 115023 10.1152/physrev.1979.59.4.863

[54] Edwards MS, Wei R, Hawes J, Sutherland-Smith M, Tang C, Li D, et al The neonatal giant panda: hand-rearing and medical management In: Wildt DE, Zhang A, Zhang H, Janssen DL, Ellis S (eds.), Giant Pandas: Biology, Veterinary Medicine and Management. New York: Cambridge University Press; 2006: 315–333.

