# Supplementary material for: Use of urinary 13,14, dihydro-15-keto-prostaglandin F2α (PGFM) concentrations to diagnose pregnancy and predict parturition in the giant panda (Ailuropoda melanolecua)
Source: PLoS One. 2018 May 2;13(5):e0195599. doi: 10.1371/journal.pone.0195599 (PMC5931454; doi:10.1371/journal.pone.0195599)
Supplement: S1 File — (PDF) [file pone.0195599.s001.pdf]

Smithsonian Conservation Biology Institute
Center for Species Survival

19 September 2017

Dr. Joerg Heber
Editor-in-Chief, *PLoS One*
Public Library of Science
1160 Battery Street
Koshland Building East, Suite 225
San Francisco, CA 94111, USA

Dear Sir:

I am writing this letter in support of Beth Robert's study on PGFM in giant pandas during pregnant and pseudopregnant cycles, and to corroborate an unpublished citation in her paper that we have conducted ultrasound on our female, and that at least once, we were unable to locate a fetus, even when completed in the days leading up to what was a successful birth. Thus, ultrasonography is not a fool-proof method to diagnose pregnancy in this species, as it requires specialized equipment, highly trained ultrasonographer, and probably more importantly, the female being in a proper position to scan the entire reproductive tract. False negative results are not uncommon.

Sincerely,

Janine L. Brown

Janine Brown, PhD
Research Physiologist/Endocrine Laboratory Head

SMITHSONIAN CONSERVATION BIOLOGY INSTITUTE
National Zoological Park
1500 Remount Road, Front Royal, VA 22630
540-635-6586 | brownjan@si.edu
www.nationalzoo.si.edu
