# Supplementary material for: Use of urinary 13,14, dihydro-15-keto-prostaglandin F2α (PGFM) concentrations to diagnose pregnancy and predict parturition in the giant panda (Ailuropoda melanolecua)
Source: PLoS One. 2018 May 2;13(5):e0195599. doi: 10.1371/journal.pone.0195599 (PMC5931454; doi:10.1371/journal.pone.0195599)
Supplement: S2 File — (PDF) [file pone.0195599.s002.pdf]

OKC ZOO
BOTANICAL GARDEN
AQUARIUM

September 25, 2017

Dear Dr. Joerg Heber:

I am writing regarding my unpublished data that are cited in a manuscript entitled "Use of urinary 13,14, dihydro-15-keto-prostaglandin F2-alpha to diagnose pregnancy in the giant panda (*Ailuropoda melanolecua*)", submitted to *PLoS One.* The manuscript states the following:

> However, pregnancy detection by ultrasonography is limited to 2-3 weeks prior to birth, requires a trained and cooperative panda during the late luteal phase when females often become unresponsive to behavioral training [9] and even when ultrasonography is performed during critical times, fetuses have gone undetected in multiple successful pregnancies (J. Brown and R. Snyder, unpublished data).

I supervised the giant panda program at Zoo Atlanta from 1999-2014. A giant panda housed at Zoo Atlanta gave birth on 6 September 2006. The mother participated in ultrasonography procedures a few times in the two to three weeks preceding the birth, but no evidence of a fetus was detected. During late pregnancy, giant pandas become very lethargic and appetite is significantly suppressed. Thus, it can be difficult to view the entire uterus during the limited time that a giant panda will cooperate with ultrasonography during this period. Additionally, the fetus is tiny compared to the size of the mother and can go undetected if the entire uterus is not visualized.

Sincerely,

Rebecca J. Snyder, Ph.D.
Curator of Conservation and Science
Oklahoma City Zoo and Botanical Garden
2000 Remington Place
Oklahoma City, OK 73111
Phone: 405-425-0256
Email: rsnyder@okczoo.org
